# Induction Treatment in Transplant-eligible Multiple Myeloma

**DOI:** 10.1097/HS9.0000000000000560

**Published:** 2021-04-26

**Authors:** J. Quinn, S. Glavey, C. Comerford, P. Murphy

**Affiliations:** 1 Department of Haematology, Beaumont Hospital, Dublin, Ireland; 2 School of Medicine, Royal College of Surgeons in Ireland (RCSI), Dublin, Ireland.

We read with great interest the recently published joint European Haematology Association and European Society for Medical Oncology clinical practice guidelines for diagnosis, treatment, and follow-up of patients with multiple myeloma (MM).^1^ With regard to induction chemotherapy in newly diagnosed MM patients who are eligible for high-dose therapy and autologous transplantation (ASCT), we note, in a change from the previous 2017 guideline, the recommendation in Figure 1 to use (if available) either daratumumab (Dara) plus bortezomib, thalidomide, and dexamethasone (Dara-VTD) or bortezomib, lenalidomide, and dexamethasone (VRD) and to use either bortezomib, cyclophosphamide, and dexamethasone (VCD) or VTD only if either of VRD or Dara-VTD are unavailable.^1,2^ As the authors point out, we know from randomized controlled trials that Dara-VTD provides superior postinduction outcomes to VTD and that while VTD showed a higher overall response rate compared with VCD (92.3% versus 83.4%), grade 3–4 peripheral neuropathy developed in 21.9% of VTD-treated patients in this study.^3,4^ However, while VCD was found to be noninferior to VRD in a randomized phase II study, neither VCD nor VTD has been directly compared with VRD in the setting of a large phase III randomized trial.^5^ The absence of such direct comparisons makes it challenging to be fully confident about the optimum “backbone” induction regimen for transplant-eligible patients, if such exists, before the anticipated widespread addition of monoclonal-antibody (MAb) therapy to induction protocols for those MM patients eligible for ASCT.

Indeed, much of the most recent evidence that informs current standard practice stems from the large EMN02, IFM2009, and CASSIOPEIA randomized studies in MM patients with de novo disease eligible for ASCT.^3,6,7^ These studies employed 3 different induction protocols: 3–4 cycles of VCD in EMN02, 3 cycles of VRD in IFM2009, and 4 cycles of VTD or Dara-VTD in CASSIOPEIA. Very good partial response rates (VGPR) or better at the end of induction were 41% in EMN02, 45% in VRD arm, and 47% in ASCT arm of IFM2009 and highest in CASSIOPEIA at 56% in the VTD control group. After ASCT, the VGPR rates were similar at 64% in EMN02, 70% in IFM2009, and 67.3% in CASSIOPEIA (VTD control group).^3,6,7^

However, differences in eligibility and patient characteristics highlight the problematic nature of cross-trial comparisons. For example, a creatinine clearance (CrCl) of >50 mL/min was required for IFM2009 enrolment, >40 mL/min for CASSIOPEIA but just >15 mL/min for EMN02. In addition, the proportion of cases with high-risk cytogenetic features was 25% in EMN02, 19.4% in IFM2009, and 16% in CASSIOPEIA with differences in the percentage of plasma cells required to exhibit a particular genetic aberration before inclusion in the high-risk group also varied between trials. For example, in the CASSIOPEIA study, >50% of plasma cells examined were required to display deletion 17p compared with >20% in EMN02. Important differences in maintenance therapy protocols are also evident. In IFM2009, lenalidomide 10 mg once daily (OD) maintenance stopped at 1 year but a dose increase to 15 mg OD was permitted, as tolerated. In comparison, lenalidomide 10 mg OD was continued till progression as tolerated with a median duration of 34.3 months maintenance therapy reported in the ASCT arm of EMN02. In addition, while there were 2 cycles of VRD consolidation in the IFM2009 study for all patients randomized to ASCT, 210 of 702 ASCT patients in EMN02 received a double-ASCT following which there was a further randomization to VRD consolidation or no consolidation. Despite the differences in induction regimens, the number of induction cycles received and subsequent trial protocols, median progression-free survival (PFS) rates in the ASCT arms in both EMN02 and IFM2009 are similar at 56.7 months and 50 months, respectively, while the CASSIOPEIA data are not mature enough to report at this point. Overall survival data are also very similar; 75.1% at 5 years in the EMN02 and 81% at 4 years in the IFM2009 studies, respectively.^3,6,7^

These outcomes deserve attention as the focus moves to monoclonal antibody (Mab)-based induction combinations. Preclinical studies have highlighted the potential role for cyclophosphamide in enhancing the anti-MM effect of daratumumab via its effects on the MM microenvironment, specifically recruiting macrophages and augmenting antibody-dependent cellular phagocytosis.^8^ These results are underscored by early clinical experience of daratumumab in combination with VCD in MM, where bortezomib was dosed weekly at 1.6 mg/m^2^. For example, a phase Ib study of Dara-VCD, delivered as induction therapy in MM patients eligible for ASCT led to an impressive post-induction overall response rate and VGPR rate of 94% and 67%, respectively.^9^ This combination is currently being compared with VTD in the EMN18 randomized study in transplant-eligible MM and additional trials have explored or are investigating daratumumab and other anti-CD38 MAbs (such as isatuximab) in combination with VRD, VTD, and KRD (K, carfilzomib).

In the meantime and outside of clinical trials, it seems that VRD is almost certainly the regimen to be most widely employed in treating transplant-eligible MM where VRD is available. However, bearing in mind the outcomes at the end of induction, post-ASCT, and PFS data in EMN02 and IFM2009, we suggest that VCD continues to represent an effective induction regimen that is very well tolerated with predictable and manageable effects on blood count parameters, being both convenient to deliver, well tolerated, and relatively inexpensive.

## Disclosures

The authors have no conflicts of interest to disclose.
